# Decision making in NICE single technological appraisals: How does NICE incorporate patient perspectives?

**DOI:** 10.1111/hex.12594

**Published:** 2017-07-07

**Authors:** Ferhana Hashem, Michael W. Calnan, Patrick R. Brown

**Affiliations:** ^1^ University of Kent Canterbury Kent UK; ^2^ University of Amsterdam Amsterdam The Netherlands

**Keywords:** decision‐making, drug appraisals, NICE, patient experts, rationing, written and oral evidence

## Abstract

**Context:**

The National Institute for Health and Care Excellence (NICE) has an explicit mandate to include patient and public involvement in the appraisal of medicines to be available for funding on the NHS. NICE involves an appraisal committee who are required to take on board experiential evidence from patient experts alongside population‐based evidence on clinical and cost‐effectiveness when making a decision whether to fund a drug.

**Objective:**

This paper considers how NICE Single Technological Appraisal (STA) committees attempt to incorporate the views of patients in making decisions about funding medicines on the NHS.

**Methods:**

A prospective design was employed to follow three pharmaceutical products involving three different appraisal committees. Three data collection methods were used: analysis of documentary evidence sent by NICE, non‐participant unstructured observations of the open and closed sessions of meetings and qualitative interviews.

**Settings and participants:**

Unstructured non‐participant observations were carried out at nine STA meetings, and 41 semi‐structured interviews were undertaken with committee members from NICE's STA committees, patient experts, analysts from NICE's project team and drug manufacturers.

**Results:**

Our analysis showed how the committees displayed a preference for an ideal‐type of patient representative, disagreement among the committee when weighing‐up patient statements in the STA process and more pre‐preparation support for patient involvement.

**Conclusions:**

Although NICE has attempted to adopt an approach flexible to patients and carers through formal decision‐making arrangements that incorporate patient views, nonetheless, the processes of the STAs can in fact undermine the very evidence collected from patient representatives.

## 1 INTRODUCTION

The role of the National Institute for Health and Care Excellence (NICE) is to improve the quality of health and social care in England, including making recommendations about the value‐for‐money of new and existing medicines. The goal behind this latter task is to assure the consistency of equitable access of drugs to patients across the entire NHS, as well as to ensure the efficient use of public finances by regulating NHS consumption of expensive drugs, and evaluate “novel” medicines against the criteria of cost‐effectiveness.^1^, ^2^ Through NICE, the Department of Health has sought to regulate the introduction of new and existing medicines and treatments within the NHS, basing its decisions on a review of clinical and economic evidence principally, at least for single technological appraisals (STAs), provided by the drug manufacturer. The advice provided by NICE is aimed at overcoming the previously ad hoc, discretionary decisions in order to standardize access to health‐care technologies across England based on evidence; however, the politicization of these decisions has resulted in NICE facing much criticism and challenges to its legitimacy.^3^, ^4^

STAs are one of NICE's decision‐making processes in which evidence about a selected technology (often medicines) is evaluated in three distinct phases (scoping, assessment and appraisal). In the last phase of this process, an independent appraisal committee evaluates evidence in a meeting (or multiple meetings), partly held in public but with the latter half taking place in a “closed” session. During the meeting(s), the committee considers evidence based on clinical and cost‐effectiveness, as well as statements expressed by patients, commissioning experts and clinical specialists.^5^ The Institute encourages patient experts attending the meeting to provide both written and oral commentary about their *personal view* in the current management of the condition and the expected role and use of the technology—in particular how it might provide benefit to patients.

### 1.1 NICE incorporating patient perspectives

Patient and public involvement (PPI) within the planning and development of health services is by no means a new concept in the domain of user involvement;^6^ in particular, PPI has in recent years in the UK become a growing feature in the organization and delivery of health care.^7^, ^8^, ^9^, ^10^ Since the mid‐1990s there have been moves towards an, “open, accountable and patient‐centred service and an attempt to establish the involvement of service users in health‐care services,” and within the UK NHS, there have been a number of initiatives giving individuals and groups a stronger voice within the health service in activities such as planning and development, extensive lobbying within hospital Trusts^11^ and involving the public in commissioning decisions and strategies.^12^ Although PPI has become increasingly prevalent, relatively little is known about which approaches work best, when or why, or under what circumstances better outcomes can be achieved making it difficult to draw conclusions about the effectiveness of different involvement approaches.^13^ One of the key aspects underpinning such uncertainty is how PPI contributes to decision making in the review and appraisal of new drug therapies. Researchers have questioned whether the arrangements for engaging and involving users in the decision‐making process are effectively addressed through formal structures set up by organizations such as NICE in their appraisals processes.^14^ This paper considers the contribution patient experts make in NICE's STA committee meetings, identifies what level of participation patient experts currently have, and what types of health‐care decision‐making contexts or domains the current model of involvement reflects.

The difficulties in incorporating patient perspectives when appraising new drugs are noted by Milewa. He explains how in one respect NICE is charged, “with the production of evidence‐based decisions…But in a second regard NICE is politicized in that it has to afford interested parties access to the decision‐making process, demonstrate cognisance of these myriad voices and reflect transparency in attendant rationales for its rulings.”^15^ Thus, NICE is required to demonstrate that it has consulted with various active specialists—not only typical experts including clinicians, health economists, NHS managers, pharmacists and researchers from health technology manufacturers, but patient groups who provide detailed witness statements. Milewa argues that inclusion of various active patient participants, “appears to constitute a more inclusive, deliberative, approach to decision making.” In pragmatic terms however, Milewa describes the difficulty in implementing these two positions, as NICE attempts to balance these two decision‐making logics—reasoning according to explicit criteria (clinical and cost‐effectiveness) and upholding an equality of status with all participants in the appraisal process. In his 2005 qualitative study, Milewa found that clinical and professional actors (as noted above) appeared to play a major role in debarring, reshaping or admitting the more subjective accounts or experientially based forms of evidence submitted by patients and carers.^16^

In the last 5 years, policy reforms in the NHS and in social care organizations denote a far greater expectation that patients, service users, carers and the public will be involved in decisions regarding the delivery of health‐care services, as reflected in four key policies: the *Health and Social Care Act (2012)*, the *NHS Constitution (2012)*, “*Putting people at the heart of care” (2009)* and “*Essential Standards of Quality and Safety” (2010)*. In line with these wider tendencies in English health policy,^17^ NICE has outlined its own key principles to promoting PPI:

- That lay people, and organizations representing their interests, have opportunities to contribute to developing NICE guidance, advice and quality standards, and support their implementation, and
- That, because of this contribution, our guidance and other products have greater focus and relevance for the people most directly affected by our recommendations.^18^

Through its 2013 policy paper on *Patient and Public Involvement Policy*, NICE's commitment to involving patients, service users, carers and the public appears integral across its range of guidance, products and quality standards documentation. In relation to STAs, NICE's continued commitment to ensuring this level of PPI appears evident. For every STA, NICE invites submissions from all patient and carer groups with interests relevant to the appraisal, and their written and oral statements must be taken on board by the committee involved in the decision making.

In the context of technological appraisals the main purpose of qualitative research is to explore areas such as patients’ experiences of having a disease or condition, their experience of having treatment and their views on the acceptability of different kinds of treatments^5^

NICE specifically outlines the areas where patient and carer perspectives would provide the most benefit to the appraisal committee; this includes consideration of both “majority views and views held by only a few patients even if they contradict the majority”; therefore, NICE appears to invite a spectrum of patient views to be taken into account, even in contexts where views may differ. In addition, NICE states that the appraisal committees are keen to incorporate patient perspectives in areas where there are gaps in published research that do not adequately capture outcomes that are important to patients, and help gauge valid clinical outcomes and standardized generic instruments in measuring quality of life to the disease or condition.^5^ Therefore, NICE and its committees might find themselves in a potentially incongruous position: how to take on board the experiential evidence from individual experts alongside evidence on clinical and cost‐effectiveness when reaching a decision about whether or not to recommend a drug as cost‐effective for NHS use.

Committee members involved in the appraisal process are selected on merit following an open and fair recruitment process where posts are publically advertised. The STA selection panel assesses candidates’ CVs and supporting letters who best meet the criteria for the role, and are then invited to interview. At interview the panel enquires about applicants’ skills and experience asking specific questions to assess if they meet the person specification. Prospective committee members are expected to have experience in health or social care as a practising health and social care professionals, or working in association with the wider aspects of health and social care. Members of the appraisal committee are selected from a spectrum of backgrounds including medical (GPs, consultants, clinicians) and non‐medical health‐care professionals (nurses and other allied health professionals), NHS managers, pharmacists, academics (epidemiologists, health economists, statisticians and public health practitioners), as well as lay experts. NICE therefore seeks to appoint members of the committees based upon their relevant experience or their specific technical skills.^19^ In terms of the patient experts asked to provide witness statements to the STA committee meetings, the Public Involvement Programme (PIP) manager contacts them in advance to explain what their role is, to complete their personal statement and prepare them for the meeting.^20^

While there is a growing emphasis on patient involvement in NICE technological appraisal meetings, nonetheless prospects for greater participation remain somewhat unclear, and therefore, the expectation and assumption of what patient experts may bring to the decision‐making process varies considerably. Charles and DeMaio present a conceptual framework based on three elements of a decision‐making domain (treatment, service delivery and broad macro‐ or system level decision‐making contexts), which incorporates different role perspectives (a user of health services, and a public policy perspective), taking into account a range of levels of participation (consultation, partnership and lay control), thus providing a step towards understanding the contribution lay participation may make in health‐care decision making. This framework helps to delineate the types and levels of lay involvement that take place at STA meetings (discussed further below), and the implications of this involvement for decision making at the meetings.^6^

In other aspects of its work, NICE appears to have a broader commitment to involving patient experts (such as expert eyewitnesses from the voluntary or community sector) through its PIP offering practical and detailed advice on how individuals can be involved in NICE's individual programmes in public health, health technologies, clinical practice or guidance products.^21^ While NICE's express commitment to incorporating PPI is evident, there have been notable barriers to patient experts’ participation in NICE technology appraisal meetings. In a survey published by NICE in 2012 of patient experts, respondents reported that the level of patient expert participation varied across different STA committees, and that their contribution to the appraisal meetings was largely dependent upon the committee Chair.^20^ Gibson and colleagues argue that in order for lay engagement to gain full recognition to complement the analytic and reductionist approaches of scientific reason, professionals, including the committee Chair, must engage in deliberations outside their traditional professional terrains. Thus, the value provided by lay experts is dependent upon the committee Chair to provide “knowledge spaces” to help establish a new terrain where experiential evidence, interpretations and opinions, and on the other hand, rigorous evaluations and scientific rationality, come together to build “creative dialogues to inform health‐care policy decisions.”^22^

Patient experts also reported that the meetings were “very large, formal and with a lot of technical language, and that the emphasis on clinical and cost‐effectiveness overshadowed patient issues.”^20^ They described not feeling able to contribute to issues of paramount importance, and they did not feel sufficiently sure or confident of their role in the meetings. Worryingly, some patient experts felt intimidated at meetings and, less surprisingly had difficulty following the presentations. The barriers to lay persons’ participation are noted by Gibson and colleagues in their examination of the concept of PPI. They observed how difficult, if not impossible it was for lay persons to achieve participatory parity in the presence of professionals. They argued that current PPI statutes may instil a semblance of equality in participation, nonetheless in specific meeting settings, “the abstract and technical forms of knowledge that professionals tend to have acquired through formal education are highly valued than the practical, experiential knowledge that patients or members of the public may possess.” Unfortunately when lay persons sit on prestigious medical committees their contribution may be unconsciously undermined, because “they do not have access to dominant forms of capital that professionals have access to.”^22^ The patient experts felt that their presence at meetings was largely “tokenistic” and that the patient experience had little to do with the committee's overall decision.^20^ The literature has emphasized such tokenism as one key reason why PPI has thus far had limited influence on the organization, planning and delivery of health‐care services, despite its emphasis in recent policy. Yet, in exploring the role of patients in NICE appraisal decision making, it is important to go beyond this narrative to consider the possibility of greater influence as well as to nuance analyses of the process of marginalization where this takes place.

## 2 METHOD

The study used a prospective design to follow three pharmaceutical products through the STA process, which were chosen for variation in the socio‐cultural resonance of the illness they were designed to treat. The final selection included: a drug treating a less “prominent” but prevalent chronic illness (Case Study X); a drug treating a high profile condition (Case Study Y); and lastly, a drug which treated a rare but life‐threatening condition (Case Study Z).^23^, ^24^

The study used an ethnographic research approach using three distinct methods of data collection: first, an analysis of documents released by NICE on each drug/therapy made available to the public online, documents submitted by the pharmaceutical company and review documents prepared by independent academic advisors. Documentary analysis was undertaken to identify points of contention, areas of uncertainty in the data, as well as providing the researchers contextual information about the drug under assessment.

Second, for each drug appraisal, semi‐structured interviews were conducted with a purposive cross‐sectional sample of the key informants (see Table 1). Forty‐one interviews, both face‐to‐face (15) and telephone (26) (three with NICE staff plus 14 in case study X, 12 in case study Y and 12 in case study Z), were carried out. Each interview lasted between 45 and 90 minutes, was voice‐recorded and was then transcribed. The semi‐structured interview guide covered topics which probed around their experiences of the meeting, how informants dealt with issues of uncertainty, whether they felt there were influences in the wider social‐cultural environment that influenced decision making, other external influences in the appraisal process on decision making, and how they found working with NICE as an organization.

**Table 1:** Participant type and number of interviews

	Patient experts	Clinical experts	NICE committee members	Drug manufacturers	Specialist assessment groups	Sub total
Case study X	1	1	8	2	2	14
Case study Y	1	1	9	1	0	12
Case study Z	1	1	6	2	2	12
NICE overview interviews	–	–	–	–	–	3
Total						41

Lastly, nine non‐participant unstructured observations of open and closed sessions of the committee meetings were carried out across the three case studies. Observational data were collected by two researchers who attended each meeting; one researcher recorded detailed discussions from the meeting verbatim, while the second researcher collected observational data on key discussions and debates, including any major points of uncertainty. It was not possible to utilize a structured observational schedule due to the technical nature of discussions. The field work was carried out between 2012 and 2014.

Three researchers were involved in the data collection activities for the documentary analysis, interviews and non‐participant observations. All three members of the research team brought with them a range of meaningful perspectives, experiences and standpoints which influenced the research process in terms of data collection and analysis (see Analysis section below).^25^

### 2.1 Patient and public involvement

The original study was funded by the Economic and Social Research Council (ESRC) on a project entitled “Managing uncertainty within NICE technological appraisals: the nature and impact of the ‘social features’ of decision‐making.” While the proposal originally set out to study difficulties in reaching decisions due to multiple logics and inputs, the specific issue of patient involvement was a theme that emerged from the data in the subsequent analysis phase. Thus, further data on how much experience the participants had of PPI in research and in the overall STA process were not covered in the study's objectives.

### 2.2 Ethical considerations

Ethical approval for the study was provided by the Ethics Committee for Research Council Grants—Social Science and Humanities, approved by the ESRC subcommittee from the University of Kent (25842). In addition, we registered the project under the University's Research Governance Framework (RGF) as the study fell under the Department of Health's RGF.

The main ethical issues encountered concerned potential identification of participants, in particular as membership of each of the committees is published on NICE's website. It was ensured that reference to the drug under appraisal, the corresponding committee and any information relating to the participants (including age, gender, years working on STAs) was removed to ensure anonymity.

### 2.3 Access and recruitment

The participating departments of NICE gave their approval for the research to be carried out. The initial approach was then made to the committee Chairs who provided an agreement by which we gained permission to undertake the non‐participatory observations; in addition, individual consent was obtained from the committee members for the closed part of the meetings.

The first contact to recruit interviewees was made face‐to‐face by the researchers who approached all potential participants including NICE committee members, as well as external contributors such as patient experts, clinical experts, members of specialist assessment groups and representatives from the pharmaceutical industry (see Table 1). Consent was taken from each of the informants.

### 2.4 Analysis

In the analysis presented below, it was considered how the committee incorporated data primarily from trials about clinical and cost‐effectiveness with experiential accounts from patients, exploring how they synthesized each type of data to reach a decision during a STA meeting on whether or not to recommend a drug for availability on the NHS. The data from the non‐participant observational field notes and transcribed informant interviews were first categorized into themes and subthemes^1^ and were then coded using NVIVO by two members of the team. Samples of the data were then double‐coded and discussed between the research team to advance reliability and construct validity. These critical discussions around the data and its interpretation also enabled a more reflexive approach where initial assumptions, or individual insights emerging from having conducted particular interviews, for example, were able to be challenged and reflected upon. Because patient involvement was not one of our initial themes, our analyses of this process were arguably less confined by clear expectations but more implicit assumptions (based on our reading around NICE) that shaped our analytical standpoints and it was important that existing arguments (for example regarding tokenism) were not simplistically reproduced. Reflexivity was further stimulated by triangulation of our data sources (documentary analysis, interviews and non‐participant observations) within the analysis, which rendered neat conclusions about one STA “reality” impossible and forced an acknowledgement of the multiple “realities” of decision making for different participants at different moments.^26^ Triangulating the official narratives of written reports with the messiness of decision making as indicated through interviews and observations illuminated processes of power while tensions between observations (what people did) and interviews (what people said they did) also helped us explore the social structures of decision making, not least the taken‐for‐granted aspects.^23^, ^27^

The analysis undertaken focused upon *manifest* themes, which were themes drawn directly from the interviews and observable content within the data set, rather than *latent* themes, which are tacit deductions, where participants refer to social distances from certain groups.^28^ Daly et al.^29^ describe thematic analysis as an emergence of themes which suitably describe the subject under investigation, and such themes can be manifest or latent, and thus, our analysis of the data is a well‐recognized approach. The data extracts available from the observations were limited, not least because the patient expert testimonies came at the latter stages of the open part of the meeting, and thus made up a relatively small part of the interactions at the meeting. The patient's input was discussed later by the committee in the closed meetings to different extents across the three cases.

## 3 RESULTS

### 3.1 Contextualizing the disease alongside the benefits of the drug

The relevance and importance of patients’ perspectives were recognized by the committee members, who generally expressed that these views were central to enable a complete appraisal of the drug technology under assessment:

… it's so amazingly helpful to have people with all different perspectives because everybody will have their own, not just perspective but their own strengths in interpreting the evidence and some people will come from, for example lay people, with a sort of common sense lay perspective…

(Committee Member)^1^

The committee member above noted the importance of having a pluralistic representation of views at the appraisal meetings as well as having a “common sense” perspective. In other respects, members of the committee thought the patient accounts helped understand the disease or condition better:

But it's really useful to have the context of the condition put in front of you… I think that's the bit where I find the patients really help with…And know, you know, what it is like day‐to‐day living with this condition…

(Committee Member)

The Chair then probes the patient experts on the significance of weight loss…A second patient expert said that a big problem is weight gain which leads to negative stereotypes and nonadherence so a drug that leads to weight loss is attractive…

(Observation notes Case Study X)

One committee member spoke about how well a patient presented herself with a specific condition, which demonstrated how the drug had improved her quality of life:

She said, “This is my quality of life so…” and she seemed quite energetic and so NICE [the STA committee] were kind of quite… quite… quite… Yeah, they were quite taken by how she presented herself etc. because, you know, she was a patient…

(Committee Member)

### 3.2 Conflicts in decision making and credibility

Alongside the usefulness of these insights, however, committee members reported that patient accounts were also an area of disagreement between members of the committee:

Because, you know, they're the people who are actually suffering and they're telling you what a huge difference it can make to their lives or the… the length of time they have left so that's sometimes quite a struggle…So it's hard… it's hard to keep… to stay neutral, I think, at times”

(Committee Member)

The informant above acknowledged that even though her role at the appraisal meeting was to provide an objective viewpoint into evaluating the evidence alongside the patient perspective; nonetheless, she admitted it was hard for her to remain neutral. She empathized with the patients and admitted that she found it hard to put personal sentiments aside. A recognition of the potential power of the patients’ narratives was also attached to a wariness among a number of committee members.

In one case study, some committee members were critical of patient experts’ contributions to the appraisal process and questioned the merits of their inclusion in meetings. One committee member spoke about the validity of including patient experts who had very little or no experience of being prescribed the medication under review:

I've seen impassioned pleas for drugs that are completely ludicrous because the person didn't have the drug that anyway the combination has been discussed, secondly they've got a particularly difficult case and they've been selected for that reason so… I don't think that the patient representative is necessarily ever very illuminating…

(Committee Member)

The comment above suggests that when patient experts were brought in to discuss the nature of the disease, rather than whether the drug had an effect, this was less than helpful and raised doubt about the credibility of the patient expert instead.

As noted above, the power and credibility of patients’ testimonies led to some misgivings about their influence.^30^ A further concern noted by several committee members was that they felt mistrustful of the patient experts due to how they were selected. Several accounts suggested that the patients were often selected via patient groups, who in turn were seen as having close links with manufacturers due to their connection with the manufacturer for example through receipt of funds from the pharmaceutical industry:

I've certainly been aware of it once where it was quite clear that a company was heavily supportive of the particular sort of patient support group and I found that quite difficult to be completely objective about…

(Committee Member)

More often than not, committee members were suspicious and disapproving of patient experts and patient representative organizations who were seen to have a close association with the drug manufacturers. Committee members also seemed to feel that patient experts’ views were too narrowly concerned with having the drug available, rather than seeing the bigger picture of rationing and the broader consequences for the NHS.

### 3.3 Tokenism

The patient experts themselves were mindful of their inclusion at the appraisal meetings and acknowledged that they were in many respects, merely representatives without a dominant presence or with any significant degree of persuasion in the final decision:

No. I… I really felt that second time I interrupted about the [name of] surgery, the patient experts really you could have almost said we… we weren't needed there

(Patient Expert)

The second meeting I…No, I didn't feel that…I didn't…I think it was slightly unusual circumstances that I was invited back to the second meeting because you wouldn't normally be invited back…And I felt that I wasn't asked many questions, which was fine but I didn't really feel I needed to be there…I don't think I really… me being there really added anything to proceedings

(Patient Expert)

The two patient experts quoted above were commenting on two separate STA appraisals, yet, both referred to feeling marginalized and surmised that their viewpoints were not being taken into account. They both questioned why they were invited to attend when they felt they “weren't needed to be there.” The first participant described feeling quite aggrieved by the way she was interrupted when asked to comment.

In contrast, another patient expert's contribution at the meeting was commended as particularly compelling as she was able to articulate in the language appropriate to the committee the problems with the disease as well as her recovery after taking the medicine (Observation notes). In an interview with this patient expert, she reflected positively about her experience and felt the committee were receptive to her account. She was also conscious that she was not a typical patient and presented herself well:

I think they listened to what I said. I think maybe in hindsight they might not have thought of me as a typical patient because I was young so that may be… in hindsight maybe we should have put forward an older person but I think, you know, the fact that I've, you know, I'm quite confident to talk to people and I know quite a lot about the condition myself anyway whereas perhaps an older patient might not always.

(Patient Expert)

In this sense, the individual characteristics and capital of the patient expert were influential, while the role of the Chair was also influential. Within our observations, the Chairs appeared conscious of the need to demonstrate publically that they welcomed the views of patient experts:

…the Chair reasserts that the committee particularly values the contribution from the lay experts as the charity suggested that if cost effectiveness is the only measure taken seriously by NICE it casts doubt on patient involvement…

(Observation notes Case Study Y)

Yet, in the closed sessions disclosed exactly what they thought of the drug:

Also the Chair felt it [the medicine] was not innovative although felt the need to mention that one of the charities saw it as ‘ground breaking’ as the committee did not want to be accused of not taking the patient experts seriously…

(Observation notes Case Study Y)

The actions of the Chair reflect how it was necessary to acknowledge the contribution of the patient experts of how they viewed the drug, in this case, as innovative. In another case, the Chair drove a discussion regarding the plausibility of the quality of life model (which initially was questioned) by advocating that the committee would be more flexible in accepting this model (even though the case was not entirely clear) as a way of reflecting the compelling arguments put forward by the patient expert.

### 3.4 More support for incorporating patient perspectives

We have already noted that the characteristics and conduct of individuals (patients or Chairs) could shape the relative influence and input of patients:

…but if anything I feel the patients are not represented as strongly….for them on their behalf and often they come in dribs and drabs and sometimes if they're not assertive enough they may not even get to say anything…

(Committee Member)

Moreover, evidence from interviews pointed towards deeper structural issues. This same committee member noted a lack of preparation was evident among the patient experts attending, with this leading to rather poor contributions and suggested that NICE should support and prepare the patient experts through the appraisal meetings:

And not to worry if they're sitting in the meeting and not understanding what's going on because there are bits that you won't understand and that type of thing. I think if they just had a patient just off the street – I'm not sure how they'd recruit one – I'd hope that they would help a little bit more.

(Patient Expert)

So while recruiting “patients off the street” would help overcome the issues of trust (and involvement with patient groups) noted earlier, our interview and observation data points to concerns about level of preparation (above) and expertise. At some moments, patient experts were asked to comment upon technical data by the committee even though their role was not to make an assessment about the drug under appraisal:

[Committee member] asks the patient expert how does a patient progress; “is this a reasonable characterisation?” he asks. [Patient expert] says that she can't comment. She states that regarding whether patients have reasonable quality of life, it depends on different patients. Patients are made aware so that they can make the decision for themselves…

(Observation notes Case Study Y)

The question posed by the committee member about patient progress was quite unexpected and the patient expert felt compelled to remind the committee that she did not have the expertise to comment, which in turn could be seen as publically devaluing her presence at the meeting.

## 4 DISCUSSION

In the analysis presented above, the aim was to consider how NICE incorporates patient perspectives within the framework of the STAs process. Although NICE provides the formal structures and processes in its appraisals for involving and engaging patient groups, the role of such groups is confined to the realm of “representation” rather than that of a key stakeholder in decision making.^14^ Of the committee members and NICE staff which were interviewed (26) across the three STA meetings, the majority recognized the contribution of patient involvement in helping to understand the condition, as well as providing a representational function for each patient group at a meeting. Although the Chair was important in acknowledging the contribution of patient views in the appraisals process, their lack of first‐hand experience of PPI diminished the importance of including witness statements in the STA decision‐making process. As commissioned by the *Breaking Boundaries Strategic Review of Public Involvement* and recommended in the final report, *Going the Extra Mile* that “a supportive, competent and influential leader [is]…critical to the successful delivery of involvement.”^31^ In particular a Chair with previous experiential knowledge of public involvement would be well positioned to champion involvement to promote changes in the institutional and organizational culture of the appraisal meetings.

Barnes et al. argue that as patient experts have been delegated a role to that solely of representation, it has caused much confusion and vagueness in terms of patient involvement. In pragmatic terms, officials have certain expectations of how patient stories should be included in the formal bureaucratic decision‐making process, and exactly what they expect patient experts to contribute to. In the case of patient involvement in the appraisals process, the model of involvement concurs with what Barnes describes as involvement on the periphery in the politics of presence, rather than a politics of patient advocacy.^32^ According to Charles and DeMaio's^6^ conceptual framework of lay participation in health‐care decision making, the patient experts’ presence was primarily consultative representing the lowest rung of lay participation providing, “an opportunity for individuals to express their views, but offers no guarantee that individual views will be taken into account.”

Evidence from this study suggested that involving patients in the STA process expanded the level of complexity the committees faced as it had increased a sense of tension or conflict between committee members. The tensions between committee members were created by some having a preference for one type of evidence over another, as well as doubt and suspicion towards the patient group, who were regarded as colluding with the drug manufacturing industry. In Milewa's^15^ examination of health technology adoption by NICE, he noted a similar theme of how in the deliberative process for drug appraisals, patients and carers were viewed as, “rarely objective with regard to the value of health technologies and some variation in opportunities given to different actors to voice their opinions in a supposedly neutral deliberative arena.” The expectation that patients should provide evidence to aid decision making in a neutral arena appeared to set the patient experts up against the committee, as patient statements were not presented in official spaces of knowledge production to be taken as robust evidence alone.^22^ Moreira^33^ argues that rationing decisions need to be more transparent, accountable and democratic, only then can uncertainties be accepted when fully explored in a process of co‐production. Organizations such as the National Institute for Health Research's INVOLVE advocate greater patient involvement in research, and as shown in *Going the Extra Mile* the salience of co‐production enables the public, researchers and health‐care professionals to work together to encourage collaboration and underline the value of people's expertise through experience in the design and delivery of research.^31^

In his examination of the role of patient organizations in health technology assessment (HTA), Moreira stipulates how there is uncertainly and a lack of theoretical understanding regarding the knowledge and expertise brought by patient representatives and organizations to HTA processes. He puts forward a model or typology of patient organizations to understand how their epistemic identities are a function of a relationship between knowledge activities and network organizations. These types of organizations are (A) robust hybrid, (B) weak hybrid, (C) weak focused or (D) robust focused. According to Moreira,^34^ using this model to underpin PPI in HTA provides a sound theoretical basis that acknowledges how different forms of engagement contribute to wider knowledge‐making; in addition, it would mean relying less on the expert‐lay boundary and opening up membership to hybrid committees where experts, practitioners and patients articulate the relationship between the evidence base and circumstances under which the technology is used.

In many respects, patient experts are tasked with performing a conflicting role where they are expected to present themselves as a credible patient while at the same time performing the role of a charismatic patient representative. In this study, it was found that patient experts who appeared at committee meetings were atypical, as they had been able to contribute to formal meeting settings, and, those invited did not reflect the archetypal patient. Patient experts who attended the meetings had two main reasons for their participation, the first as a patient sufferer with the condition or disease, then as the enigmatic storyteller or marketer advocating the patient group. In these circumstances, the committee placed the patient experts in a position where they foresaw their contribution as an aid to reaching a decision in a neutral public forum, but when presented with a patient statement, they found their impassioned accounts too emotional or hard to handle.^35^

Evidence from this study did show that the overriding priority of the committee was to appraise the technology in terms of cost‐effectiveness and the extent to which it fell in or outside the threshold set by NICE.^24^ In one of our cases, the technology was far too expensive and a decision not to recommend was made after two meetings which may explain why there was limited patient expert involvement. However, in the other two cases, there was considerable more uncertainty about the incremental cost‐effectiveness ratio and possibly therefore more space for patient expert influence.

Notwithstanding the study showed that throughout the process for all three cases, social influences on decision making were both explicit and implicit, suggesting that the discourse on cost‐effectiveness was overriding and apparent in formally documented meetings, but contributions from patient experts also had a tacit impact on the appraisal throwing open debate between committee members around rigorous evidence vs patient witness statements. This study also only focused on three of the four appraisal committees, although there is no reason why the one not included should adopt a different approach to the importance of incorporating patient expert perspectives.

Although NICE has implemented some pioneering initiatives to incorporate patient perspectives, these initiatives have left some patient groups feeling that their role at the STAs has been largely peripheral and perhaps even tokenistic. Some patient representatives questioned whether they would accept an invitation to attend another meeting in the future. Patient experts described feeling unsupported in their attendance at meetings and were in some instances dismayed that other lay experts would not be able to contribute to meetings given the technical nature of discussions.^20^ NICE's attempts to engage with patients experts in STAs have helped to exclude the very groups the appraisal system has intended to involve. While NICE declares values and aspirations appearing to be open, transparent, participatory and pluralist in its involvement structure, yet in practice, the process is a closely managed, unitary system and a top‐down model, where salaried professionals run a highly regulated public participation process. Their approach to public involvement has been predominantly technicist or an instrumental approach, without any overriding concern for a pluralist involvement structure where a diversity of views can be publically articulated.^22^

It has been shown that the role of patient representatives at appraisal meetings differed considerably—while some patient experts were described as helping the committee understand the disease/condition, in other respects they were also seen as advocates of the new drug therapy. It would appear that NICE needs to provide much greater guidance and clarity over the roles and contributions it expects patients to make and how their statements and submissions might fit into the decision‐making framework.^6^ The blueprint for positive outcomes and greater impact for PPI is recommended in the RAPPORT study by Wilson et al. who put forward six salient actions for effective PPI, the first of which is aimed at defining the purpose, role and structure of PPI. Having a clearly defined role is paramount for NICE to be able to carve out a bespoke function for patient experts, to understand where in the decision‐making framework patient experts reside and what purpose their contribution makes at the appraisal meetings and within wider STA deliberations.^36^

## 5 CONCLUSIONS

The evidence from this study has shown that patient experts have provided a symbolic and representative function at committee meetings where the patient voice is relegated to the periphery of participation. The formal appraisal processes and structures set up by NICE still appear to fall short of creating knowledge spaces where committee members weigh up different types of evidence both experiential accounts alongside rigorous evidence about clinical and cost‐effectiveness. The results show that despite laying out an explicit commitment to incorporating patient perspectives through its PPI policy, the arrangements for engaging and involving users through its deliberative processes in fact marginalize the very groups it has sought inclusion for at STA appraisal meetings.

## CONFLICT OF INTEREST

The above authors declare no conflict of interests.

## Note

1 We have withheld reference to each specific case study for the interviews with informants in order to preserve anonymity.
